# Supplementary material for: Complexome profiling of the Chlamydomonas psb28 mutant reveals TEF5 as an early PSII assembly factor
Source: Plant Cell. 2025 Mar 17;37(6):koaf055. doi: 10.1093/plcell/koaf055 (PMC12164590; doi:10.1093/plcell/koaf055)
Supplement: koaf055_Supplementary_Data [file koaf055_supplementary_data.zip › psb28_interactive.zip!/plots/intensity/qualityPlots/peptides/FAP287_Cre06.g260400.t1.2.html]

### Cre06.g260400.t1.2

Trivial name: FAP287  
SeqCoverage [median]: 21.30  
Mass: 13.41 kDa  
  
 

| Sequence | MissedCleavage | Mass [Da] | wtIntensity | muIntensity | Score |
| --- | --- | --- | --- | --- | --- |
| AQPAPAKPSAGATPPQK | 1 | 1615.87 | 579.76 | 0.00 | 35.33 |
| MEEETISIR | 0 | 1106.53 | 2528.82 | 4308.25 | 110.87 |
|  |  |  | 3108.6 | 4308.3 |  |
